# Utilizing Geographical Distribution Statistical Data to Improve Zero-Shot Species Recognition

**DOI:** 10.3390/ani14121716

**Published:** 2024-06-07

**Authors:** Lei Liu, Boxun Han, Feixiang Chen, Chao Mou, Fu Xu

**Affiliations:** 1 School of Information Science and Technology, Beijing Forestry University, Beijing 100083, China; liulei815@bjfu.edu.cn (L.L.);; 2 Engineering Research Center for Forestry-Oriented Intelligent Information Processing of National Forestry and Grassland Administration, Beijing 100083, China; 3 State Key Laboratory of Efficient Production of Forest Resources, Beijing 100083, China

**Keywords:** CLIP, zero-shot classification, species recognition, geographical distribution statistical data

## Abstract

**Simple Summary:**

Species recognition is a key part of understanding biodiversity and can help us to better conserve and manage biodiversity. Traditional species recognition methods require large amounts of image data to train the recognition model, but obtaining image data of rare and endangered species is a challenge. However, Contrastive Language–Image Pre-training (CLIP), a generalized artificial intelligence model, can perform classification by calculating the similarity between images and text without the need for training data. Taking advantage of this and considering the unique geographic distribution pattern of species, we propose a CLIP-based species recognition method that can recognize species based on geographic distribution knowledge. This study is the first to combine geographic distribution knowledge with species recognition, which can lead to more effective recognition of rare and endangered species.

**Abstract:**

Species recognition is a crucial part of understanding the abundance and distribution of various organisms and is important for biodiversity conservation and management. Traditional vision-based deep learning-driven species recognition requires large amounts of well-labeled, high-quality image data, the collection of which is challenging for rare and endangered species. In addition, recognition methods designed based on specific species have poor generalization ability and are difficult to adapt to new species recognition scenarios. To address these issues, zero-shot species recognition based on Contrastive Language–Image Pre-training (CLIP) has become a research hotspot. However, previous studies have primarily utilized visual descriptive information and taxonomic information of species to improve zero-shot recognition performance, and the use of geographic distribution characteristics of species to improve zero-shot recognition performance has not been explored. To fill this gap, we proposed a CLIP-driven zero-shot species recognition method that incorporates knowledge of the geographic distribution of species. First, we designed three prompts based on the species geographic distribution statistical data. Then, the latitude and longitude coordinate information attached to each image in the species dataset was converted into addresses, and they were integrated together to form the geographical distribution knowledge of each species. Finally, species recognition results were derived by calculating the similarity after acquiring features by the trained CLIP image encoder and text encoder. We conducted extensive experiments on multiple species datasets from the iNaturalist 2021 dataset, where the zero-shot recognition accuracies of mammals, mollusks, reptiles, amphibians, birds, and insects were 44.96%, 15.27%, 17.51%, 9.47%, 28.35%, and 7.03%, an improvement of 2.07%, 0.48%, 0.35%, 1.12%, 1.64%, and 0.61%, respectively, as compared to CLIP with default prompt. The experimental results show that the fusion of geographic distribution statistical data can effectively improve the performance of zero-shot species recognition, which provides a new way to utilize species domain knowledge.

## 1. Introduction

Biodiversity is essential for maintaining the stability of natural ecosystems [1,2,3]. Understanding the distribution and abundance of various organisms is essential for biodiversity conservation and management [4]. Species recognition is one of the crucial steps in achieving this goal. With the rapid development of artificial intelligence, automatic species recognition methods based on deep learning have become more and more popular [5,6,7,8,9,10].

However, species recognition methods based on traditional vision-only deep learning models face two challenges. Firstly, they usually require a large amount of labeled image data for training, which may make it difficult to obtain a large amount of high-quality image data for some rare and endangered species. And labeling these data requires trained animal experts, which is time-consuming and laborious. Secondly, the models have poor generalization ability and cannot make full use of previously learned knowledge when facing problems such as new datasets, few-shot learning, or zero-shot learning.

In recent years, Vision–Language Models (VLMs) such as Bootstrapping Language–Image Pretraining (BLIP) [11] and Contrastive Language–Image Pretraining (CLIP) [12] have been pretrained on large-scale datasets for general-purpose tasks and have learned rich multimodal information, which allows them to be used for downstream tasks with little or no training. The emergence of these foundational models provides an insight into zero-shot and few-shot learning for a variety of downstream tasks, such as anomaly localization in industrial detection [13], image geolocalization [14,15], point cloud understanding [16], biomedical tasks [17,18], and so on. Similarly, there are many zero-shot recognition methods based on CLIP for species recognition. Guo et al. [19] proposed CALIP, which allows text and image features to interact with each other to improve the accuracy of zero-shot species recognition. Zhou et al. [20] and Shu et al. [21] both utilized the strategy of test-time enhancement to improve the performance of zero-shot species recognition. Maniparambil et al. [22] utilized visual descriptions as prompts to improve the CLIP-based zero-shot recognition accuracy on the CUB dataset [23] by 2.73%, compared to the CLIP-based zero-shot recognition accuracy of 54.70% with the default prompts. Parashar et al. [24] improved the CLIP-based zero-shot species recognition accuracy from 9.21% to 20.17% on the semi-iNaturalist dataset [25] by converting the scientific names of species to common names. Stevens et al. [26] developed BioCLIP, which makes full use of the taxonomic information of the species to improve the zero-shot recognition accuracy on the Insects dataset [27] to 34.8%, compared to the CLIP-based zero-shot recognition accuracy of 9.1%.

In addition to morphological characteristics and taxonomic relationships, species data include attributes such as habitat, ecological behavior, and geographic range. Species are distributed in different geographic regions due to climate, topography, vegetation types, and other factors. For instance, giant pandas are mainly found in Sichuan, Shaanxi, and Gansu Provinces in China [28], while amur tigers are mainly found in northeastern China and Russia [29]. Utilizing the geographical distribution information of species can provide more comprehensive cognitive background and contextual information for species recognition models. Although previous studies [30,31,32] have fused images and structured geolocation information together through training to improve species recognition performance, it is difficult to apply to zero-shot species recognition without training. Considering that structured geolocation information messages can be converted into knowledge of geographical distribution in textual form, this provides a vision for Vision–Language Pretraining models to utilize species geographic distribution knowledge to improve species recognition performance. However, this has not yet been explored.

To fill this gap, we proposed a CLIP-driven zero-shot species recognition method which can utilize knowledge of the geographical distribution of species to improve the accuracy of zero-sample species recognition. Firstly, we transformed the latitude and longitude information accompanying each species image in the dataset into an address to obtain statistical knowledge of the geographical distribution at the country or province level. Secondly, we constructed textual prompts by combining the scientific or common name of the species with the species geographical distribution statistical data. Finally, we used CLIP’s image encoder and text encoder to extract features from the images and textual prompts, respectively, and by calculating the similarity between the two, we obtained the results of zero-shot species recognition.

The three contributions of our study are as follows:

- We proposed a CLIP-driven zero-shot recognition method that takes the geographical distribution statistical data of species as a prompt for the first time, which provides a new perspective on the use of biological domain knowledge to improve species recognition performance.
- We further explored the performance of different administrative levels of geographical distribution statistical data for zero-shot species recognition.
- We demonstrated the effectiveness of the proposed method on multiple species superclass subsets in the iNaturalist dataset [33] under different weather conditions and lighting conditions.
- Our proposed method exhibited significant advantages over other state-of-the-art methods and was able to complement them.

## 2. Methods

**Figure 1:**
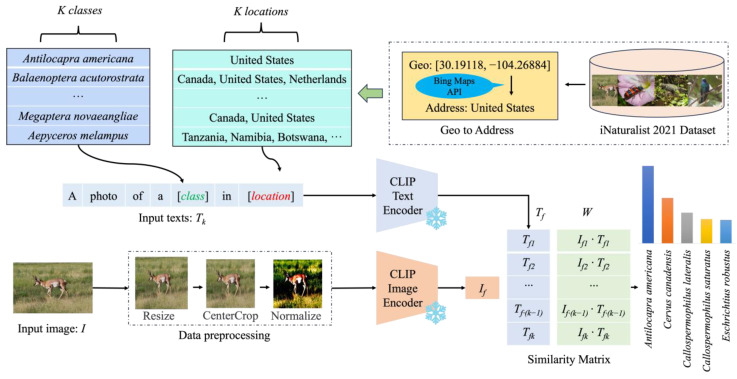
The structure of the zero-shot species recognition model based on geographical distribution statistical data. CLIP denotes Contrastive Language–Image Pre-training, an image–text pretraining model that contains a text encoder and an image encoder that can be used to extract image and text features.

In this section, we first introduce CLIP, describing its process for performing zero-shot classification on a new species dataset. Then, we present prompt design methods that incorporate the geographical distribution statistical data of species. Finally, the process of acquiring geographical distribution statistical data for a species is described, and a prompt design method that incorporates these geographical distribution statistical data is presented. The overall framework of our proposed method is shown in Figure 1. The pseudocode of the proposed method is shown in Algorithm 1.
**Algorithm 1**: Zero-shot species recognition method utilizing geographical distribution statistical data**Input**: Species names *S_K_*; Geographical distribution statistical data of each species *G_k_*; Prompt template *PT*; *m* test species images *I_m_*

**Output**: Ranked species list for test images *P_m_*1 *T_k_* ← PT(*S_K_*, *G_k_*)2 *T_f_* ← TextEncoder(*T_k_*)3 **for** *i* = 1 to *m* **do**4    *I_f_* ← ImageEncoder(*I_i_*)5    *W* ← *T_f_* · *I_f_*6    *p_i_* ← Softmax(*W*)7    *P_m_* ← *p_i_*8 **end for**9 **return** *P_m_*

### 2.1. CLIP-Driven Zero-Shot Species Recognition

CLIP, a large-scale vision–language model consisting of an image encoder and a text encoder, obtained by minimizing contrast loss and trained on 400 million image–text pairs collected from the Internet, is the most representative model for unsupervised learning, and has shown great potential in learning relocatable representations, successfully achieving zero-shot or few-shot recognition in a range of visual classification tasks.

Here, we have presented the workflow of CLIP for zero-shot species recognition. CLIP consisted of an image encoder and a text encoder. The image encoder contained two different architectures, including ResNet [34] and Vision Transformer (ViT) [35]. The text encoder used the Transformer [36] architecture. Specifically, for given a species image $I\in R^{H\times W\times C}$, *H*, *W*, and *C* denote the height, width, and number of channels. According to Equation (1), the species image *I* was converted to image features $I_{f}\in R^{D}$ after passing through an image encoder, where *D* denotes the dimension of the image features.

(1) $$I_{f}=ImageEncoder\left(I\right).$$

Given *K* species, the default prompt template “A photo of a [class]” of CLIP was used to generate sentences *T_K_* for *K* species, where class is the species name. Then, the text features of each species *T_f_* were obtained after implementing a text encoder, according to Equation (2).

(2) $$T_{f}=TextEncoder\left(T_{K}\right).$$

Finally, according to Equation (3), the corresponding similarity matrix $W\in R^{D\times 1}$ was calculated using the text features and image features, where *D* is the dimensions of the image. Then, the similarity matrix was used for the *Softmax* function to obtain the maximum probability value *p*, which is the corresponding species.

(3) $$p=Softmax\left(W\right).$$

### 2.2. Prompts Design That Incorporate the Geographical Distribution Knowledge of Species

To utilize CLIP for zero-shot species recognition, it was critical to design appropriate prompts. However, designing prompts manually for different datasets required significant experimental and time costs. CoOp [37] and CoCoOp [38] proposed learnable prompts that allow a model to automatically learn corresponding prompts from data after few-shot training, but the learned prompts cannot be interpreted. For a species, it varied in appearance, range, habitat environment, habits, and so on. Taxonomists can use such differences to recognize species. Therefore, we utilized the attributes of the species itself to design prompts to improve the performance of CLIP-driven zero-shot recognition. In this paper, we chose species geographic distribution knowledge to design suitable prompts and proposed a prompt template “A photo of a [class] in [location]” that incorporates knowledge of geographic distribution to generate text for different species. This can reflect the geographic distribution patterns of different species, allowing their geographic distribution patterns to adapt to the visual feature space.

In taxonomy, the scientific names of species such as mammals, reptiles, birds, etc., consist of Latin, Greek, etc., and are highly specialized concepts. Based on the species’ scientific name and its geographical distribution knowledge, we proposed the first prompt “A photo of a [scientific name] in [location]”.

In addition, in order to popularize the knowledge of species to the public and to deepen the understanding of nature, species have common names. Therefore, we designed the second prompt “A photo of a [common name] in [location]” by replacing the scientific name with the common name of the species.

Due to cultural and geographical differences, the same common name may correspond to different species. Therefore, we further designed the last prompt “A photo of a [scientific name, common name] in [location]” by combining the scientific and common names of the species.

### 2.3. Acquisition of the Geographical Distribution Knowledge of Species

The geographical distribution knowledge of species is usually updated and refined through field surveys, specimen collection, satellite remote sensing, and so on. However, the number of species and their numbers are so large that obtaining knowledge of their geographic parts is very time-consuming. Here, we utilized an extremely simple way to obtain knowledge of species’ geographic distribution. First, using the geographic location information attached to each species image in the dataset, the latitude and longitude coordinates were converted to the actual geographic location through Bing Maps’ API. Second, after the de-duplication operation, statistical data on the geographical distribution of species could be obtained at different administrative levels, e.g., country and province levels. Based on the above species geographic distribution statistical data and the prompt template in Section 2.2, for different species datasets, we could generate corresponding sentence prompts for zero-shot species recognition. Then, we performed zero-shot species recognition according to the process in Section 2.1.

## 3. Experiments

### 3.1. Datasets

Most species datasets contain only image data with relatively simple image backgrounds and fewer species classes. To investigate the impact of fusing geographic distribution statistical data on the recognition performance of zero-shot species under complex background conditions, we used the iNatutalist 2021 dataset to validate our proposed method. This dataset was initially used for fine-grained species classification and contained 10,000 classes collected from around the world. Each image on the iNaturalist 2021 dataset contains latitude, longitude, and time of day. For zero-shot species recognition, we only used its validation set. We selected six superclasses from this validation set to produce six sub-datasets, including Mammals, Mollusks, Reptiles, Amphibians, Birds, and Insects. The final number of species categories in the 6 datasets was 4910, encompassing the vast majority of species categories. Details of the validation set in the iNaturalist 2021 dataset that we used are shown in Table 1.

**Figure 2:**
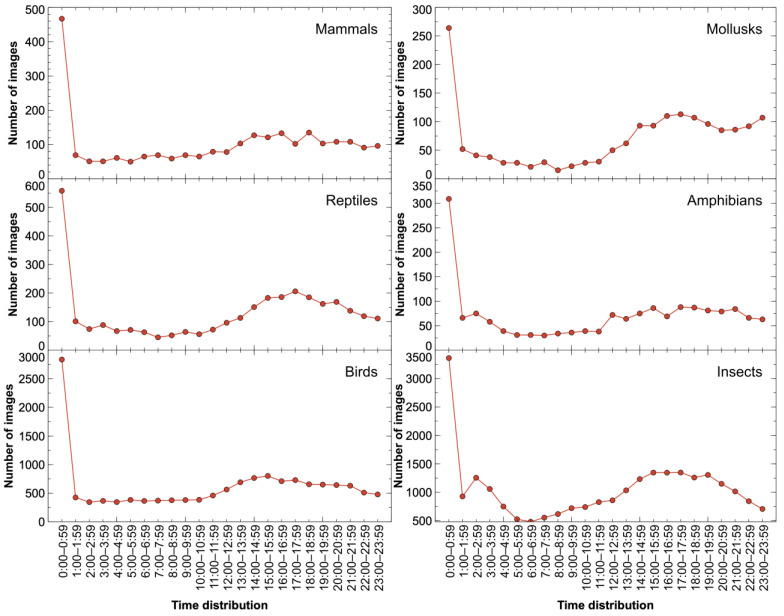
Time distribution in the six species superclass datasets. There is an anomaly in the number of images in the range of 0:00–0:59 on each sub-dataset due to the lack of specific time for part of the images.

Further, we counted the time distribution on the six sub-datasets based on the time when each image was taken. As can be seen in Figure 2, the number of images in each sub-dataset had a different temporal distribution, covering different lighting conditions, including day and night.

**Figure 3:**
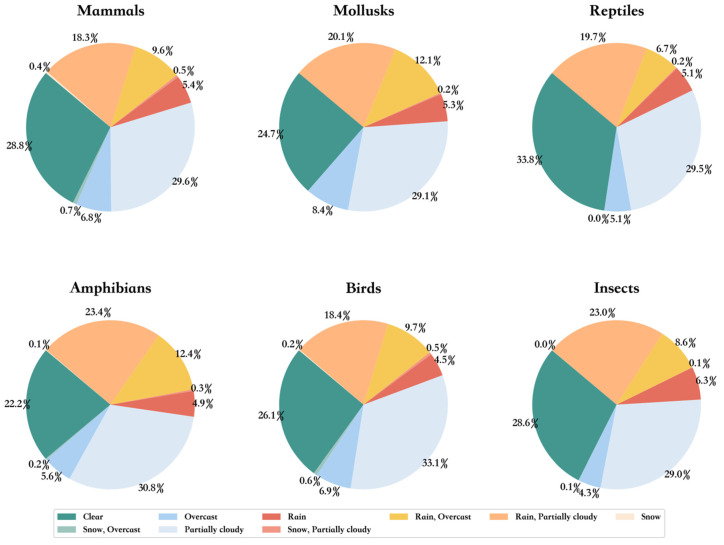
Weather distribution in the six species superclass datasets. Weather information was obtained through the Historical Weather API in Visual Crossing.

Next, we obtained the weather conditions of the day based on the latitude, longitude, and time information of each image. As can be seen in Figure 3, the images on each sub-dataset involve a variety of weather conditions, including rain, snow, overcast, and clear.

### 3.2. Experiment Settings and Evaluation Metrics

We implemented all methods on an NVIDIA GeForce RTX 3090 GPU (NVIDIA, Santa Clara, CA, USA) with 24 GB using the framework Pytorch 1.7.1 and the programming language Python 3.8.0. We conducted comparative experiments on three CLIP ViT backbones, including ViT-B/32, ViT-B/16, and ViT-L/14. The configuration of the different versions of CLIP’s image encoder is shown in Table 2.

In the validation phase, we pre-processed each image, including resizing to 256 × 256 and center-cropping to 224 × 224, and then normalized it according to the mean (0.485, 0.456, 0.406) and standard deviation (0.229, 0.224, 0.225). We used Accuracy, Precision, Recall, and F1-Score as evaluation metrics to evaluate the effectiveness of our proposed method.

## 4. Results

In this section, we first verified that fusing the knowledge of species’ geographical distribution can improve the zero-shot species recognition performance. Secondly, we verified the effect of different visual encoders of CLIP on the zero-shot species recognition performance of fusing species’ geographical distribution statistical data; and then, we further explored the effect of different administrative levels of the species on species recognition. Finally, we compared our proposed method with CALIP [19], Prompt Ensemble [12], and TPT [21].

### 4.1. Comparison Experiment with CLIP’s Default Prompt

Using ViT-B/32 as an image encoder for CLIP, we compared the performance of our prompt designed by uniting species’ scientific name and geographic distribution statistical data with the default prompt of CLIP. As shown in Table 3, zero-shot species recognition accuracy increased by 0.7% on average after fusing the geographic distribution statistical data in six different species datasets. Zero-shot species recognition accuracy increased in five datasets, Mammals, Reptiles, Amphibians, Birds, and Insects. Zero-shot species recognition accuracy decreased in Mollusks. The largest improvement in accuracy was 2.4% for Mammals and the smallest was 0.09% for Insects.

### 4.2. Comparative Experiments with Different Species Name Representations

To validate the performance of fusing geographic distribution statistical data under different species name representations for zero-shot recognition, we compared the three prompts presented in Section 2.2 with the default prompt of CLIP. CLIP’s image encoder was ViT-B/32. The experimental results are shown in Table 4.

For Reptiles, under the three prompts fusing geographic distribution statistical data, the zero-shot species recognition accuracy was improved by 0.89%, 0.38%, and 0.77%. For Birds, under the three prompts fusing geographic distribution statistical data, the zero-shot species recognition accuracy was improved by 0.5%, 0.62%, and 0.36%.

For Mammals, the zero-shot species recognition accuracy was 32.15% when using the prompt designed with the species’ common name. When using the scientific name and geographic distribution statistical data as the prompt, or when using the scientific name, common name, and geographic distribution statistical data as prompts, the zero-shot species recognition accuracy was improved by 2.4% and 0.32%, respectively. When using the common name and geographic distribution statistical data as prompts, the zero-shot species recognition accuracy decreased by 0.93%. For Amphibians, when using the scientific name and geographic distribution statistical data as prompts, or when using the scientific name, common name, and geographic distribution statistical data as prompts, the zero-shot species recognition accuracy was improved by 0.94% and 0.24%, respectively. When using the common name and geographic distribution statistical data as prompts, the zero-shot species recognition accuracy decreased by 0.44%. For Insects, when using the scientific name and geographic distribution statistical data as prompts, the zero-shot species recognition accuracy was improved by 0.09%. When using the common name and geographic distribution statistical data as prompts, or when using the scientific name, common name, and geographic distribution statistical data as prompts, the zero-shot species recognition accuracy decreased by 0.05% and 0.16%, respectively.

For Mollusks, under the three prompts fusing geographic distribution statistical data, the zero-shot species recognition accuracy decreased by 0.6%, 0.35%, and 0.39%.

### 4.3. Comparative Experiments with Different CLIP Image Encoders

We compared the performance of zero-shot recognition using different CLIP image encoders, when using species’ scientific name, common name, and geographical distribution statistical data as prompts. As shown in Table 5, as the image encoder changed from ViT-B/32 to ViT-L/14, the ability of the model to extract image features became stronger, and the semantic associations between image features and textual features became clearer, resulting in better and better zero-shot species recognition. When the image encoder was ViT-B/32, incorporating geographic distribution statistical data improved the zero-shot recognition performance of Mammals, Reptiles, Amphibians, and Birds by 0.32%, 0.77%, 0.24%, and 0.36%, respectively. When the image encoder was ViT-B/16, incorporating geographic distribution statistical data improved the zero-shot recognition performance by 1.1%, 0.28%, 0.17%, and 0.22% for Mammals, Reptiles, Amphibians, and Insects, respectively. When the image encoder was ViT-L/14, incorporating geographic distribution statistical data improved the zero-shot recognition performance by 2.07%, 0.48%, 0.35%, 1.12%, 1.64%, and 0.61% for Mammals, Mollusk, Reptiles, Amphibians, Birds, and Insects, respectively. It can also be seen from Table 5 that under ViT-L/14, our proposed method achieved the highest Precision, Recall, and F1-Score on the six species datasets.

**Figure 4:**
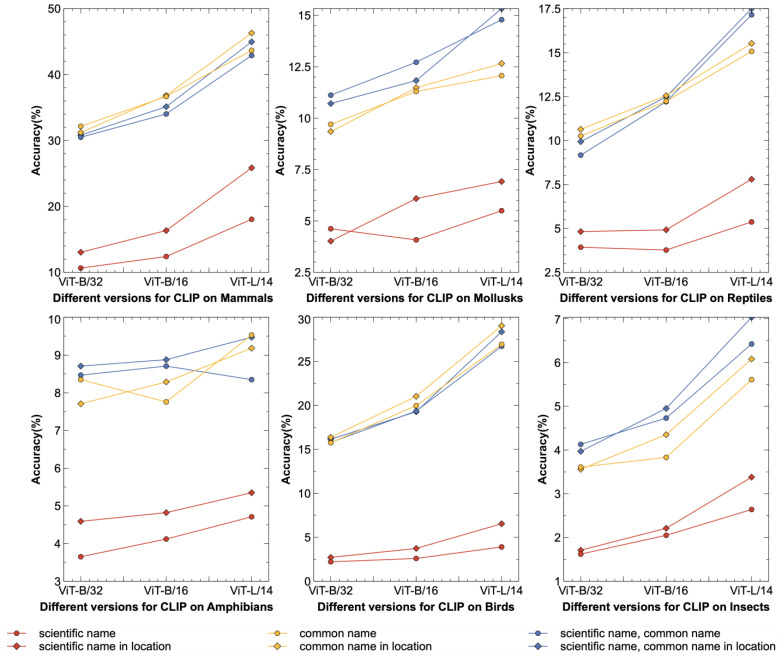
Performance analysis of zero-shot species recognition with different species datasets, different prompts, and different image encoder versions. The horizontal coordinates represent the image encoder and the vertical coordinates represent the recognition accuracy. The six subplots from top to bottom and left to right are Mammals, Mollusks, Reptiles, Amphibians, Birds, and Insects. Geographic distribution statistical data are at the national level. The prompt prefix “A photo of a” is omitted. CLIP denotes Contrastive Language–Image Pre-training.

Figure 4 details the performance of zero-shot species recognition with various species datasets, image encoders, and prompts. As the image encoder became larger, the overall zero-shot recognition accuracy was progressively better with the three prompts that fused geographic distribution statistical data compared to the prompts without fused geographic distribution statistical data.

### 4.4. Comparative Experiments with Different Administrative Levels of Geographical Distribution Statistical Data

Further, we investigated the effect of different administrative levels of geographical distribution statistical data, such as the two levels of country and province, on the recognition performance of zero-shot species. Based on the experimental results in Section 4.3, we chose ViT-L/14 as the image encoder for our experiments. Due to the relatively large number of species categories in Birds and Insects, we chose one family each from them as new datasets: Anatidae and Nymphalidae. The Anatidae dataset contained 90 species with 900 images. The Nymphalidae dataset contained 234 species with 2340 images. Based on Section 2.3, we developed geographic distribution statistical data for these species at both the country and province level.

**Figure 5:**
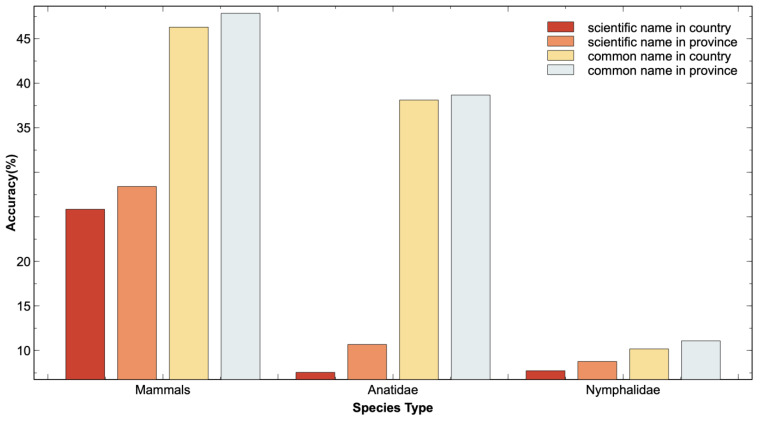
Further analysis of zero-shot recognition performance at different administrative levels of geographic distribution statistical data. The image encoder of Contrastive Language–Image Pre-training (CLIP) is ViT-L/14. The prompt prefix “A photo of a” is omitted.

As shown in Figure 5, as the administrative level of geographic distribution statistical data became deeper, i.e., the more accurate the species’ geographic range, the better the overall performance of zero-shot species recognition with the three prompts. For three datasets, the recognition accuracy increased as the geographic distribution statistical data changed from the national level to the province level with all prompts.

### 4.5. Comparative Experiments with State-of-the-Art Methods

To further demonstrate the effectiveness of our proposed method, we first compared it with CALIP. CALIP interacted with visual and textual features extracted by CLIP via a parameter-free attention module to enhance CLIP’s zero-shot recognition performance; it was used as baseline model. As shown in Table 6, it could be seen that the performance of our proposed method was overall significantly better than that of CALIP. Under ViT-L/14, compared to CLIP with the default prompt, the zero-shot species recognition accuracy of CALIP on Mammals was improved by 0.36%, while the zero-shot species recognition accuracy of our proposed method on Mammals was improved by 7.8%. More importantly, our proposed method can also be combined with CALIP to achieve better zero-shot species recognition performance. Specifically, under ViT-L/14, the performance of our proposed method combined with CALIP was 26.14%, 7.4%, 8.05%, 5.71%, 6.65%, and 3.55% on the six datasets, respectively.

In addition, we compared our proposed method with other advanced zero-shot recognition methods such as Prompt Ensemble and TPT. Prompt Ensemble uses 80 hand-crafted prompts to conduct zero-shot image classification. TPT is a test-time prompt tuning method that learns adaptive prompts with a single test sample. As shown in Table 7, our proposed method exceeds CLIP with the default prompts and Prompt Ensemble on all four species datasets. On Mammals, our proposed method achieved the best zero-shot recognition accuracy of 44.96%. On Mollusks and Amphibians, TPT achieved the best zero-shot recognition accuracy of 17.04% and 9.94%, respectively, followed by our proposed method and CALIP. On Reptiles, CALIP achieved the best zero-shot recognition accuracy of 17.8%. However, our proposed method can be used in combination with CALIP and TPT to further improve the zero-shot recognition accuracy. Specifically, the zero-shot recognition accuracies on Mammals, Mollusks, and Amphibians were 45.41%, 15.62%, and 10.35%, respectively, when CALIP and our proposed method were used in combination. When TPT was used in combination with our proposed method, the accuracy of zero-shot recognition on Mammals, Reptiles, and Amphibians was 47.64%, 19.01%, and 10.47%, respectively.

### 4.6. Comparative Experiments on Inference Time

In this section, we compared the single-image inference times on different datasets with different prompts under ViT-L/14. As shown in Table 8, when fusing species’ scientific names and geographic distribution statistical data as a prompt, there is a slight increase in inference time for a single image on the six species datasets. The average inference time per image increased by 2.02%. However, this increase is not significant and is acceptable.

### 4.7. Case Studies

To further elaborate on the effectiveness of our proposed method, we executed three case studies. First, we performed two types of case studies on Mammals. One category was where the probability of a previously correctly recognized species increased after incorporating geographic distribution statistical data. One category was where species that were not correctly recognized were correctly recognized after incorporating geographic distribution statistical data. Then, we performed case studies on subsets of the Mammals and Birds datasets to demonstrate the practical feasibility of our proposed approach.

#### 4.7.1. Increased Probability of Correct Recognition

**Figure 6:**
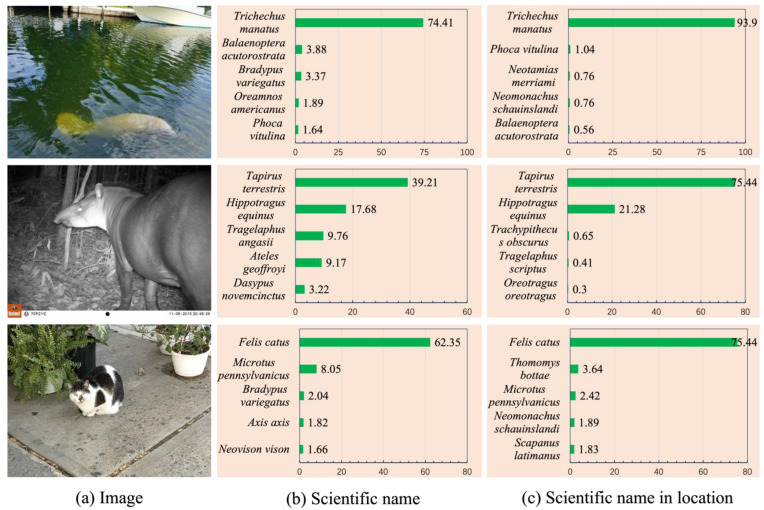
Three examples of increased probability of correct recognition. Contrastive Language–Image Pre-training’s (CLIP’s) image encoder is ViT-L/14. The prompt prefix “A photo of” is omitted. The top 5 predictions are shown for each species.

As shown in Figure 6, the probability of correct recognition of *Trichechus manatus*, *Tapirus terrestris*, and *Felis catus* increased and the probability of incorrect recognition decreased after incorporating geographic distribution statistical data at the country level. Among them, the probability of correct recognition of *Trichechus manatus* changed from 74.41% to 93.9%, an improvement of 19.59%; the probability of correct recognition for *Tapirus terrestris* changed from 39.21% to 75.44%, an improvement of 36.23%; the probability of correct recognition for *Felis catus* changed from 62.35% to 75.44%, an improvement of 13.09%.

#### 4.7.2. Change from Wrong to Correct Recognition Results

**Figure 7:**
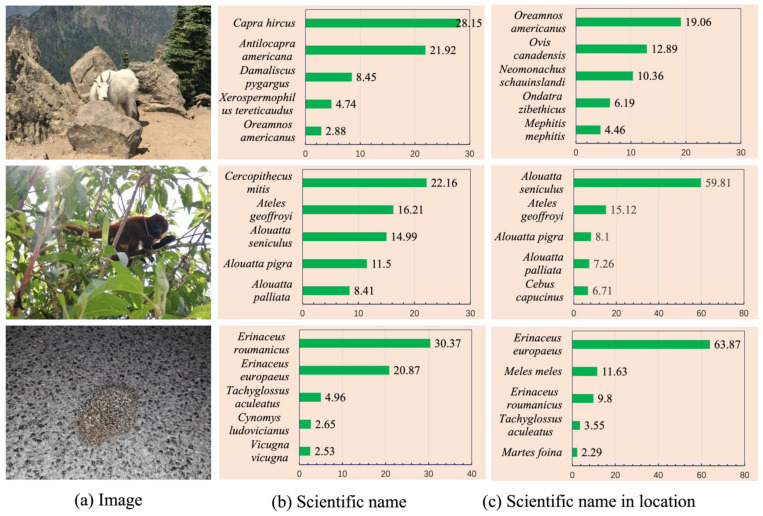
Three examples of incorrect to correct recognition. Contrastive Language–Image Pre-training’s (CLIP’s) image encoder is still ViT-L/14. The prompt prefix “A photo of a” is omitted. The top 5 predictions are shown for each species. The distribution statistics of *Capra hircus* include the United States, Gambia, Italy, China, Greece, Spain, and Indonesia; the distribution statistics of *Oreamnos americanus* include the United States and Canada. The distribution statistics of *Cercopithecus mitis* include Uganda, South Africa, Tanzania, and Kenya. The distribution statistics of *Alouatta seniculus* include Ecuador, Colombia, Venezuela, and Peru. The distribution statistics of *Erinaceus roumanicus* include Greece, Albania, Lithuania, Slovakia, Czechia, and Russia. The distribution statistics of *Erinaceus europaeus* include New Zealand, the United Kingdom, Russia, Portugal, Italy, and France. The geographic distribution statistics of these species were obtained by converting the latitude and longitude of the pictures taken in the dataset into addresses.

Figure 7 shows three examples of changes from incorrect to correct recognition after fusing geographic distribution statistical data. For the image in the first row of Figure 7a, when using only the species scientific name as a prompt, the recognition was *Capra hircusi*; after combining this with geographic distribution statistical data, the recognition changed to *Oreamnos americanus*. Based on the geographic distribution statistical data of the two species, it can be seen that *Capra hircusi* has a wider distribution range, while *Oreamnos americanus* is mainly concentrated in the United States and Canada. For the image in the second row of Figure 7a, the recognition result was *Cercopithecus mitis* when only the species scientific name was used as a prompt, and after combining this with geographic distribution statistical data, the recognition result was changed to *Alouatta seniculus*, which has a completely different range, as can be seen from the geographic distribution statistical data of the two species. For the image in the third row of Figure 7a, when only the species scientific name was used as a prompt, the top two predicted results for incorrect recognition included the correctly predicted result, and the difference between the two was 9.5%. After using geographic distribution statistical data, the difference became 54.07%.

#### 4.7.3. Comparative Experiments on Subsets of Mammals and Birds

To demonstrate that our proposed method can be applied in practice, we conducted comparative experiments on subsets of Mammals and Birds. We took the top 20 species from Mammals to create the Mammals-20 dataset, and the top 10 species from Birds to create the Birds-10 dataset. The results in Table 9 show that when there were fewer species categories, the zero-shot species recognition accuracies of CLIP with the default prompt were 69.5% and 47% on the Mammals-20 and Birds-10 datasets, respectively. When fused with geographic distribution statistics, the zero-shot recognition accuracies further improved to 71.5% and 53%. When combined with CALIP, the zero-shot recognition accuracies were 73.5% and 57%, respectively. We believe that such zero-shot recognition performance can be used in practical species recognition.

## 5. Discussion

In this paper, we confirmed that incorporating geographic distribution knowledge can improve CLIP-driven zero-shot species recognition performance.

Large-scale visual language pretraining models such as CLIP can be used in downstream zero-shot species recognition tasks due to their excellent textual and image representation capabilities. Among them, prompt engineering was the key to improving zero-shot recognition performance. To design appropriate prompts to improve the performance of zero-shot and few-shot species recognition, both Maniparambil et al. [22] and Mou et al. [39] designed prompts using species’ visual descriptions to improve the performance of few-shot species recognition. Parashar et al. [24] analyzed the dataset for training CLIP and found that the scientific name of the species appeared less often than the common name, and therefore designed prompts by converting the scientific name of the species to the common name. Stevens et al. [26] designed prompts by using the unique characteristics of the organisms, such as family, genus, and species, to retrain BIOCLIP.

The above research mainly utilized species’ visual descriptions and taxonomic attributes. Considering the uniqueness of the geographical distribution of species, incorporating knowledge of geographic distribution into species recognition models can help to better categorize species into the correct classes. Previous studies [30,32] have incorporated structured data like latitude and longitude coordinates into image-only species recognition models to improve recognition performance. However, structured data like latitude and longitude need to be trained along with the images to aid in species recognition. In the case of zero-shot species recognition processes, there is no training process and it is difficult to utilize such structured data directly. Since CLIP combines both image and text modalities, it provides a way to directly utilize the knowledge of geographic distribution to improve the performance of species recognition models. Motivated by the above research, we explored the effect of incorporating the geographical distribution knowledge of species on the recognition performance of zero-shot species. Our proposed method differs from the previous research in two ways: One is that we directly utilized knowledge of geographical distribution in the textual form rather than structured latitude and longitude information. The second is that our method does not require training and does not introduce additional parameters.

First, as shown in Table 3, we demonstrated an overall improvement in zero-shot recognition performance when using species’ scientific name and geographic distribution knowledge as prompts, with an average improvement of 0.7% on the six species datasets, with accuracy decreasing by only 0.6% on the Mollusks datasets.

Second, we investigated the effect of incorporating geographic distribution statistical data under different species name representations on zero-shot recognition performance. Table 4 indicates that under different name representations, fusing geographic distribution knowledge as a prompt improves zero-shot species recognition accuracy.

Then, we investigated the effect of fused geographic distribution statistical data on zero-shot recognition performance under different CLIP image encoders. As shown in Table 5, as the image encoder becomes more capable of extracting features, the fused geographic distribution statistical data lead to better improvements in the performance of zero-shot species recognition.

Further, we investigated the effect of different administrative levels of geographic distribution statistical data on zero-shot recognition performance. Figure 5 shows that the more accurate the geographic distribution range, the better the zero-shot species recognition performance.

In addition, we compared our proposed method with a set of advanced methods. From Table 6, we found that our proposed method was not only superior to CALIP, but can also be used in combination with CALIP to achieve better performance. From Table 7, we found that our proposed method exceeded Prompt Ensemble and CLIP with the default prompt on four datasets, demonstrating competitive performance. Meanwhile, our proposed method can also be used with other advanced methods such as CALIP and TPT to further improve zero-shot recognition accuracy.

Finally, we performed three case studies. As shown, Figure 6 and Figure 7 demonstrate how the inclusion of geographic distribution statistical data can improve the performance of zero-shot species recognition, both in terms of increased probability of correctness and in terms of turning errors into correct recognitions. As can be seen in Table 9, our proposed zero-shot recognition method performs better when there are fewer species categories and can be used in practice.

In the real world, our proposed method can be applied in education and biodiversity research, especially when the amount of rare and endangered species image data is small and it is difficult to train suitable models for accurate recognition. In scenarios where data on rare and endangered species are limited, our proposed method can improve the accuracy of zero-shot recognition of species by using records of species geographic distribution from the existing species literature and community websites. For species that are visually similar or photographed under poor lighting conditions, our proposed method can also take advantage of the uniqueness of the species’ geographic distribution to improve the accuracy of zero-shot recognition.

Since our proposed method is based on CLIP, which is a large model with large parameters, the computational resources required for deployment are considerable. In the real world, our model is suitable to be deployed in scenarios with abundant computational resources, such as data centers, desktop computers, and laptops. While in forest environments, lacking communication conditions and sufficient computational resources, offline lightweight models are needed, which can hardly meet the deployment requirements of our proposed model.

However, there are two limitations to our study. One is that for migratory birds and species with a wide global distribution, incorporating geographic distribution statistical data has a limited, if not negative, effect on species recognition performance. The second is that there is a data bias due to the fact that our knowledge of geographic distribution comes from information incidental to the images in the dataset.

In the future, we will work on two aspects. One is to find a more appropriate way to acquire geographic distribution knowledge. For instance, geographic distribution statistical data can be acquired from the Global Biodiversity Information Facility (GBIF) or community websites such as iNaturalist. Likewise, geographic distribution knowledge of species can be obtained from websites such as Wikipedia. However, rapid and accurate access to this knowledge of geographic distribution requires further study. The other is to fuse more fine-grained knowledge of species geographic distribution to design prompts and to study the incremental improvement in species recognition based on the connection between different levels of geographic distribution knowledge.

## 6. Conclusions

In this paper, we proposed a CLIP-driven zero-shot species recognition method based on species geographical distribution knowledge. We first obtained geographical distribution statistical data of species from the latitude and longitude information accompanying the images in the dataset. Then, three different prompts for zero-shot species recognition were devised jointly with species’ scientific name, common name, and geographic distribution statistical data. We executed a number of experiments on multiple species datasets and demonstrated that incorporating species’ geographic distribution statistical data improves the performance of zero-shot recognition. Specifically, the zero-shot recognition accuracies of Mammals, Mollusks, Reptiles, Amphibians, Birds, and Insects improved by 2.07%, 0.48%, 0.35%, 1.12%, 1.64%, and 0.61%, respectively, when guided by prompts designed to unite species’ scientific name, common name, and geographic distribution statistical data. Our proposed method can provide a new concept for species recognition using domain knowledge.

## Figures and Tables

**Table 1:** Details of the validation set in the iNaturalist 2021 dataset.

Sub-Datasets	Number of Species	Number of Images
Mammals	246	2460
Mollusks	169	1690
Reptiles	313	3130
Amphibians	170	1700
Birds	1486	14,860
Insects	2526	25,260
Total	4910	49,100

**Table 2:** The configuration of the different versions of the Contrastive Language–Image Pre-training’s (CLIP’s) image encoder.

CLIP’s Image Encoder	Configuration	
	Params (M)	Model Size (MB)
ViT-B/32	151.28	354
ViT-B/16	149.62	350.8
ViT-L/14	427.62	932.8

**Table 3:** Zero-shot species recognition accuracies with Contrastive Language–Image Pre-training’s (CLIP’s) default prompts and our proposed first prompts in six species datasets. The first prompts are “A photo of a [scientific name] in [location]”. The image encoder of CLIP is ViT-B/32. Geographic distribution statistical data are at the national level. Bold indicates the best species recognition performance.

Prompts	Mammals	Mollusks	Reptiles	Amphibians	Birds	Insects	Average
A photo of a [scientific name] (CLIP’s default prompt)	10.65%	**4.62%**	3.93%	3.65%	2.21%	1.62%	4.45%
A photo of a [scientific name] in [location]	**13.05%**	4.02%	**4.82%**	**4.59%**	**2.71%**	**1.71%**	**5.15%**
Improvement	2.4%	−0.6%	0.89%	0.94%	0.5%	0.09%	0.7%

**Table 4:** Zero-shot species recognition accuracies with Contrastive Language–Image Pre-training’s (CLIP’s) default prompt and our proposed three prompts in six species datasets. The image encoder of CLIP is ViT-B/32. Geographic distribution statistical data are at the national level. Bold indicates the best species recognition performance.

Prompts	Mammals	Mollusks	Reptiles	Amphibians	Birds	Insects
A photo of a [scientific name]	10.65%	4.62%	3.93%	3.65%	2.21%	1.62%
A photo of a [scientific name] in [location]	13.05%	4.02%	4.82%	4.59%	2.71%	1.71%
A photo of a [common name]	**32.15%**	9.7%	10.26%	8.35%	15.75%	3.61%
A photo of a [common name] in [location]	31.22%	9.35%	**10.64%**	7.71%	**16.37%**	3.56%
A photo of a [scientific name, common name]	30.49%	**11.12%**	9.17%	8.47%	15.79%	**4.13%**
A photo of a [scientific name, common name] in [location]	30.81%	10.71%	9.94%	**8.71%**	16.15%	3.97%

**Table 5:** Zero-shot species recognition performance with different image encoders. Geographic distribution statistical data are at the national level. Bold indicates the best species recognition performance. CLIP denotes Contrastive Language–Image Pre-training.

CLIP’s Image Encoder	Datasets	Prompts	Accuracy	Precision	Recall	F1-Score
ViT-B/32	Mammals	A photo of a [scientific name, common name]	30.49%	33.48%	30.57%	28.98%
		A photo of a [scientific name, common name] in [location]	30.81%	34.63%	30.85%	29.08%
	Mollusks	A photo of a [scientific name, common name]	11.12%	10.00%	11.07%	9.08%
		A photo of a [scientific name, common name] in [location]	10.71%	10.77%	10.71%	8.70%
	Reptiles	A photo of a [scientific name, common name]	9.17%	10.33%	8.98%	7.95%
		A photo of a [scientific name, common name] in [location]	9.94%	11.16%	9.87%	8.56%
	Amphibians	A photo of a [scientific name, common name]	8.47%	8.51%	8.47%	7.08%
		A photo of a [scientific name, common name] in [location]	8.71%	8.68%	8.71%	7.13%
	Birds	A photo of a [scientific name, common name]	15.79%	17.77%	15.81%	14.69%
		A photo of a [scientific name, common name] in [location]	16.16%	19.13%	16.18%	14.88%
	Insects	A photo of a [scientific name, common name]	4.13%	3.69%	4.11%	3.02%
		A photo of a [scientific name, common name] in [location]	3.97%	3.90%	3.97%	3.00%
ViT-B/16	Mammals	A photo of a [scientific name, common name]	34.02%	36.54%	34.07%	31.95%
		A photo of a [scientific name, common name] in [location]	35.12%	38.81%	35.08%	33.29%
	Mollusks	A photo of a [scientific name, common name]	12.72%	11.80%	12.78%	10.01%
		A photo of a [scientific name, common name] in [location]	11.83%	12.32%	11.83%	9.54%
	Reptiles	A photo of a [scientific name, common name]	12.11%	12.01%	12.08%	10.23%
		A photo of a [scientific name, common name] in [location]	12.49%	15.83%	12.56%	11.39%
	Amphibians	A photo of a [scientific name, common name]	8.71%	10.30%	8.71%	7.62%
		A photo of a [scientific name, common name] in [location]	8.88%	11.68%	8.88%	7.96%
	Birds	A photo of a [scientific name, common name]	19.36%	22.45%	19.37%	18.19%
		A photo of a [scientific name, common name] in [location]	19.28%	23.54%	19.30%	18.18%
	Insects	A photo of a [scientific name, common name]	4.73%	4.22%	4.73%	3.51%
		A photo of a [scientific name, common name] in [location]	4.95%	4.80%	4.93%	3.74%
ViT-L/14	Mammals	A photo of a [scientific name, common name]	42.89%	47.28%	42.89%	40.71%
		A photo of a [scientific name, common name] in [location]	**44.96%**	**51.39%**	**45.00%**	**43.48%**
	Mollusks	A photo of a [scientific name, common name]	14.79%	15.12%	14.79%	12.10%
		A photo of a [scientific name, common name] in [location]	**15.27%**	**18.18%**	**15.21%**	**13.48%**
	Reptiles	A photo of a [scientific name, common name]	17.16%	19.51%	17.19%	**15.89%**
		A photo of a [scientific name, common name] in [location]	**17.51**%	**21.92%**	**17.51%**	15.80%
	Amphibians	A photo of a [scientific name, common name]	8.35%	12.52%	8.41%	7.71%
		A photo of a [scientific name, common name] in [location]	**9.47%**	**12.71%**	**9.41%**	**8.77%**
	Birds	A photo of a [scientific name, common name]	26.71%	30.52%	26.72%	25.44%
		A photo of a [scientific name, common name] in [location]	**28.35%**	**33.45%**	**28.37%**	**26.75%**
	Insects	A photo of a [scientific name, common name]	6.42%	7.19%	6.41%	5.35%
		A photo of a [scientific name, common name] in [location]	**7.03%**	**8.03%**	**7.03%**	**5.82%**

**Table 6:** Comparative experiments with CALIP. Geographic distribution statistical data are at the national level. Bold indicates the best species recognition performance. CLIP denotes Contrastive Language–Image Pre-training.

CLIP’s Image Encoder	Methods	Prompts	Mammals	Mollusks	Reptiles	Amphibians	Birds	Insects
ViT-B/32	CLIP	A photo of a [scientific name]	10.65%	4.62%	3.93%	3.65%	2.21%	1.62%
	CALIP		10.93%	4.85%	4.12%	4.12%	2.33%	1.7%
	CLIP	A photo of a [scientific name] in [location]	13.05%	4.02%	4.82%	4.59%	2.71%	1.71%
	CALIP		13.41%	4.26%	5.18%	4.94%	2.75%	1.75%
ViT-B/16	CLIP	A photo of a [scientific name]	12.4%	4.08%	3.77%	4.12%	2.59%	2.05%
	CALIP		12.6%	4.62%	4.09%	4.41%	2.61%	2.13%
	CLIP	A photo of a [scientific name] in [location]	16.34%	6.09%	4.92%	4.82%	3.73%	2.21%
	CALIP		16.79%	6.21%	5.05%	5.29%	3.8%	2.31%
ViT-L/14	CLIP	A photo of a [scientific name]	18.05%	5.5%	5.37%	4.71%	3.9%	2.64%
	CALIP		18.41%	5.62%	5.75%	5.24%	4.01%	2.81%
	CLIP	A photo of a [scientific name] in [location]	25.85%	6.92%	7.8%	5.35%	6.54%	3.38%
	CALIP		**26.14%**	**7.4%**	**8.05%**	**5.71%**	**6.65%**	**3.55%**

**Table 7:** Comparative experiments with other advanced methods. The image encoder of Contrastive Language–Image Pre-training (CLIP) is ViT-L/14. Bold indicates the best species recognition performance.

Methods	Prompts	Mammals	Mollusks	Reptiles	Amphibians
CLIP	A photo of a [scientific name, common name]	42.89%	14.79%	17.16%	8.35%
Prompt Ensemble	80 hand-crafted prompts ([scientific name, common name])	43.13%	15.15%	16.87%	9.18%
CALIP	A photo of a [scientific name, common name]	43.62%	15.27%	17.8%	9.47%
TPT	A photo of a [scientific name, common name]	43.94%	**17.04%**	17.64%	9.94%
Ours	A photo of a [scientific name, common name] in [location]	44.96%	15.27%	17.51%	9.47%
CALIP + Ours	A photo of a [scientific name, common name] in [location]	45.41%	15.62%	17.64%	10.35%
TPT + Ours	A photo of a [scientific name, common name] in [location]	**47.64%**	16.51%	**19.01%**	**10.47%**

**Table 8:** Average inference time for a single image with different prompts. The image encoder of Contrastive Language–Image Pre-training (CLIP) is ViT-L/14. Geographic distribution statistical data are at the national level.

Prompts	Hardware Device	Mammals	Mollusks	Reptiles	Amphibians	Birds	Insects
A photo of a [scientific name] (CLIP’s default prompt)	GPU	0.008958 s	0.009760 s	0.008673 s	0.010286 s	0.006963 s	0.006799 s
A photo of a [scientific name] in [location]	GPU	0.009273 s	0.010280 s	0.008745 s	0.010354 s	0.007020 s	0.006808 s

**Table 9:** The accuracy of zero-shot recognition on Mammals-20 and Birds-10 datasets. The image encoder of Contrastive Language–Image Pre-training (CLIP) is ViT-L/14. Geographic distribution statistical data are at the national level.

Prompts	Mammals-20	Birds-10
A photo of a [common name] (CLIP’s default prompt)	69.5%	47%
A photo of a [common name] in [location] (Ours)	71.5%	53%
CALIP	70.5%	51%
CALIP + Ours	73.5%	57%

## Data Availability

Data are contained within the article.
